# The disease-modifying effect of dehydroepiandrosterone in different stages of experimentally induced osteoarthritis: a histomorphometric study

**DOI:** 10.1186/s12891-015-0595-1

**Published:** 2015-07-31

**Authors:** Kai Huang, Jia-peng Bao, Gavin James Jennings, Li-dong Wu

**Affiliations:** Department of Orthopedics, The Second Affiliated Hospital of Zhejiang University, Zhejiang University, Hangzhou, China; Department of Orthopedics, Tongde Hospital of Zhejiang Province, Hangzhou, China; Department of Orthopedics, Royal United Hospital Bath, Bath, United Kingdom

**Keywords:** Dehydroepiandrosterone, Osteoarthritis, Cartilage, Histomorphometry

## Abstract

**Background:**

Osteoarthritis (OA) is likely to become an increasing burden in the coming decades. Various agents have been developed to slow the progression of OA, and are collectively known as ‘disease-modifying drugs’, however, there is still little reliable evidence that such agents will be successful. Dehydroepiandrosterone (DHEA), a sex hormone precursor, has been recently proven as protective agent against OA, but the exact mechanism is still unkown. In the current study, the effects of weekly intra-articular injections of DHEA in preventing the progression of existing cartilage degeneration in an OA rabbit model were evaluated. The aim of the current study is to demonstrate the feature of its disease-modifying efficacy during OA progression.

**Methods:**

Thirty male New Zealand white rabbits were used in this study. An anterior cruciate ligament transection (ACLT) model was used to create a progressive OA model in twenty rabbits. The animals were treated with DHEA or a placebo and were necropsied at 9 and 16 weeks. Ten rabbits receiving sham operations served as controls. The articular cartilage of the medial femoral condyle (MFC), lateral femoral condyle (LFC), medial tibial plateau (MTP) and lateral tibial plateau (LTP) was evaluated macroscopically and histologically.

**Results:**

In the joints of the sham-operated rabbits, few histological changes were detected on the articular surfaces of the femoral condyles and tibial plateaus. ACLT obviously induced erosive changes on the cartilage surfaces. Compared to the placebo group, the macroscopic and Mankin score analyses demonstrated that the DHEA treatment markedly reduced the cartilage lesions and delayed cartilage degeneration in the four regions of the knee at 9 weeks after operation (macroscopic score: MFC P = 0.013; LFC P = 0.048; MTP P = 0.045; LTP P = 0.02, Mankin score: MFC P = 0.012; LFC P = 0.034; MTP P = 0.016; LTP P = 0.002). At 16 weeks, DHEA demonstrated chondroprotective effects on the lateral compartment of the knee compared to the placebo group, whereas the cartilage degeneration at the medial compartment of the knee did not differ among the groups (macroscopic score: LFC P = 0.046; LTP = 0.034, Mankin score: LFC P = 0.005; LTP P = 0.002).

**Conclusion:**

The disease-modifying efficacy of DHEA aganist OA is time-specific and site-dependent. DHEA could be used as a disease-modifying strategy to limit the progression of OA, especially in the middle stage.

## Background

Osteoarthritis (OA) is a degenerative joint disease that is generally characterized by progressive cartilage deterioration, sclerosis of the underlying subchondral bone, osteophyte formation and low-grade synovitis [1]. An imbalance between the biosynthesis and the degradation of the matrix components leads to progressive tissue destruction, resulting in extensive articular damage [2]. No disease-modifying treatment for OA exists. The available treatments for OA predominantly target symptomatic relief without addressing the fundamental mechanism underlying OA, which is the destruction of articular cartilage [3]. More studies are identifying and characterizing potential disease-modifying therapeutic agents for OA, including calcitonin [4], bone morphogenetic protein-7 [5], lubricin [6], trichostatin A [7] and mesenchymal stem cells [8].

Dehydroepiandrosterone (DHEA) is a 19-carbon steroid hormone that is classified as an adrenal androgen. DHEA has been shown to antagonize the catabolic mediators of cartilage that may exert protective effects in OA, including suppressing matrix metalloproteinases (MMPs) and inducing cartilage restoration [9]. Our recent research [10] showed that DHEA demonstrated beneficial effects on OA by influencing the balance between the aggrecanases and tissue inhibitors of metalloproteinase-3 (TIMP-3) in cartilage tissues, suggesting that DHEA might protect articular cartilage from degeneration at the molecular level. In this study, we further explored how this agent exerts its disease-modifying effect at the histomorphometric level in an identical animal model of OA.

Increasing evidence demonstrates that there are different stages of OA, each with its own distinct pattern of initiation and progression. One treatment might work in one stage of the disease and not work in another stage, which highlights the need for testing cartilage with possible intervention strategies that target existing morphological changes that occur during the different stages of OA. In this study, we explore the effect of DHEA on cartilage histology during two OA stages.

To test the reparative efficiency of DHEA, we generated a rabbit anterior cruciate ligament transection (ACLT) model of OA, in which the characteristics of the cartilage degenerative process are highly similar to those of humans. This study differs from previous research that used unilateral knee surgery as a model because in this study, the ACLT surgery was performed bilaterally to avoid compensatory locomotion in the rabbits. To investigate the histological modifying effect of DHEA in more detail, the four compartments of the knee joint, including the medial femoral condyle (MFC), lateral femoral condyle (LFC), medial tibial plateau (MTP) and lateral tibial plateau (LTP), in two stages of OA were analyzed.

## Methods

### Ethics statement

This study was carried out in strict accordance with the recommendations in the Guide for the Care and Use of Laboratory Animals of Zhejiang University. The protocol was approved by the Committee on the Ethics of Animal Experiments of Zhejiang University. All surgery was performed under sodium pentobarbital anesthesia, and all efforts were made to minimize pain and suffering.

### Reagents

DHEA (Sigma, Fluka, St. Louis, MO) was used at a concentration of 100 μmol/L (dissolved in 0.9 % normal saline [NS]). This concentration exhibited a protective effect against articular cartilage loss and demonstrated no toxic effects on the chondrocytes [9].

### Animal groups and treatment

Inclusion criteria: Male New Zealand white rabbits (one year old) weighed about 2.5-3.5 kg were included. Exclusion criteria: The knee joints of all rabbits were X-rayed before inclusion. The rabbits with clinical or radiographic evidence of joint disease were excluded. Finally, thirty rabbits met the inclusion standard and were enrolled in the current study for subsequent assessment.

In our previous study, we found that rabbits showed considerable individual variability in OA progression after ACLT. To more strictly examine the effect of DHEA on disease progression, a matched-pair analysis was used in this study. For the matched pair analyses of cartilage degradation, 20 rabbits with 40 knees were used. An additional 10 rabbits with 20 knees underwent sham surgery and served as the controls.

Twenty rabbits were intravenously anaesthetized with 3 % sodium pentobarbital (1 mL/kg). Both legs were prepared and draped in the usual sterile fashion, and an approximately 3-cm midline incision was made over the knee. After dissection through the subcutaneous tissue, the capsule was incised with a median parapatellar incision. The patella was dislocated laterally, exposing the ACL, which was then completely transected. An intraoperative Lachman test was performed to verify that instability had been created. An additional 10 rabbits received sham surgery in which the keen joint capsule was opened without transecting the ACL after luxation of patella with the same procedure as OA model. After surgery, the rabbits were provided appropriate postoperative care and analgesics. Each ACLT-treated rabbit received 0.3 mL of DHEA at a concentration of 100 μmol/L once a week in the left knee, and the right knee was injected with the same volume of NS as a placebo. The intra-articular interfering began at week 5. Each sham-operated rabbit received an identical volume of NS in both knees beginning at week 5. Half of the animals were randomly sacrificed 9 weeks after surgery. The remaining animals were sacrificed 16 weeks after surgery. All knees were harvested for the analyses. According to the above treatment strategy, all harvested knees were divided into the following six groups: 1) sham-operated knees harvested 9 weeks after surgery (Sham, 9 w), 2) sham-operated knees harvested 16 weeks after surgery (Sham, 16 w), 3) OA knees with normal saline treatment 9 weeks after ACLT (ACLT + NS, 9 w), 4) OA knees with normal saline treatment 16 weeks after ACLT (ACLT + NS, 16 w), 5) OA knees with DHEA treatment 9 weeks after ACLT (ACLT+ DHEA, 9 w) and 6) OA knees with DHEA treatment 16 weeks after ACLT (ACLT + DHEA, 16 w).

### Gross observation

After the rabbits were sacrificed, gross morphologic grading of the four sections (MFC, LFC, MTP and LTP) was performed separately according to the following scale: grade 0, smooth surface with normal color; grade 1, rough surface with minimal fibrillation or a slight yellowish discoloration; grade 2, cartilage erosion extending into the superficial or middle layers; grade 3, cartilage ulceration extending into the deep layers; and grade 4, cartilage depletion with the subchondral bone exposed [11]. In the current study, cartilage with grade 2–3 changes was recognized as the morphologic features of middle stage of disease process. Significant cartilage thinning, subchondral sclerosis, and osteophyte formation which were corresponding to grade 4 were considered to be the morphologic features of advanced stage of OA. The examination was performed by two independent pathologists who were blinded to the treatment groups. The specimens were photographed with a high-resolution digital camera (PowerShot G12, Canon, Japan).

### Histological examinations

**Fig. 1:**
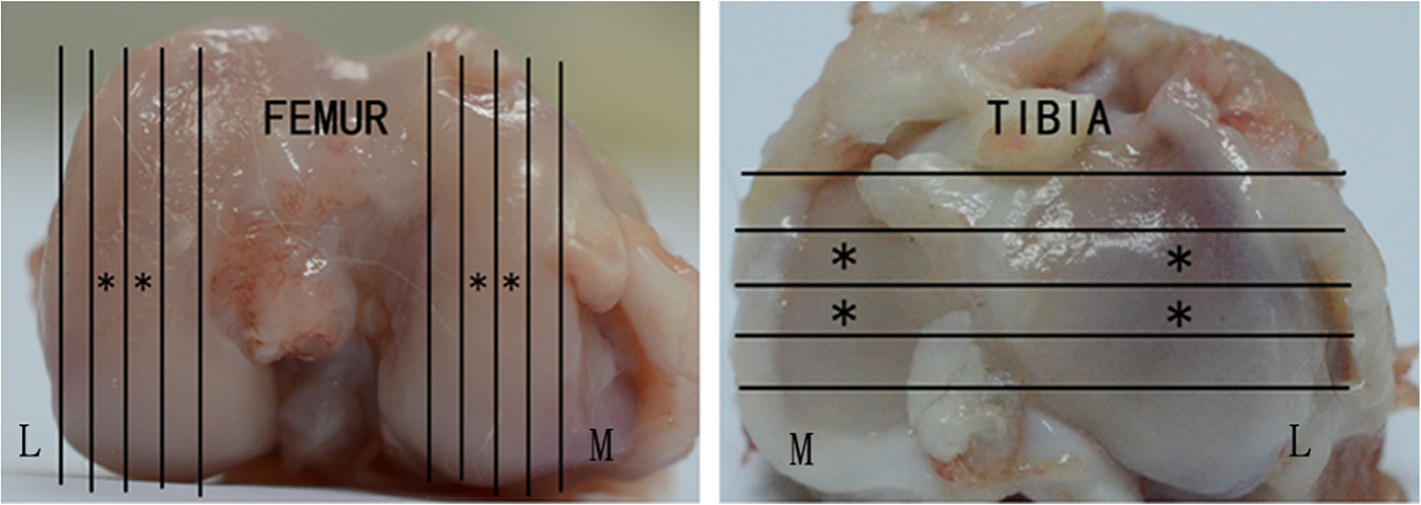
The medial and lateral tibias as well as the medial and lateral femoral condyles were each divided into four 3 mm-thick osteochondral slabs, and the central two slabs (*) obtained from each of four major weight-bearing compartments were histologically examined. M: medial. L: lateral

The proximal tibia and the distal femur of the operated knees from each rabbit were fixed in 10 % neutral-buffered formalin and then placed in 15 % disodium-ethylenediaminetetraacetic acid (EDTA-2Na). After decalcification was complete, the surface areas of the four compartments (i.e., LFC, MFC, LTP and MTP) at risk for OA lesions were each divided into four 3-mm thick osteochondral slabs (anterior to posterior for the tibia and abaxial to axial for the femoral condyle). After routine histologic processing, each osteochondral slab was embedded in paraffin (anterior surface down for the tibial slabs and abaxial surface down for the femoral condyle slabs) and 5-μm sections were stained with Safranin O. The tissue sections obtained from the two central osteochondral slabs of each four weight-bearing compartments, which usually shows the earliest and most severe histological abnormalities [12] were evaluated using the Mankin’s grading system by an experienced cartilage pathologist [13] (Fig. 1). The observer was blinded for the treatment received by the rabbit group source and macroscopic description of the samples, which were presented in random order.

### Statistical analysis

All data are expressed as the mean ± standard deviation (SD). Statistical analysis of the gross morphology data was performed using the nonparametric Mann–Whitney U test. The histological data were analyzed using a paired t-test. The statistical analyses were performed with SPSS 19.0 for Windows software, and P < 0.05 indicated statistical significance.

## Results

### Model development

All operations were performed without complications. At euthanasia, no significant variation in the body weights between the experimental groups was observed and the complete transection of each ACL was confirmed grossly. In the current study, at 4 weeks after model establishment, safranin O staining in the ACLT group was partially lost and fibroses were observed on the surface of hyaline cartilage, indicating loss of articular cartilage proteoglycan and induction of early OA. The ACLT animals in placebo group that were sacrificed at 9 weeks demonstrated histological features that included extensive partial thickness fissures, loss of the superficial zone layer and empty chondrocyte lacunae within the remaining cartilage with reduced Safranin-O staining, which is similar to the pathological changes in the middle stage of human OA. The animals that were sacrificed at 16 weeks had progressive osteophytosis in the medial knee compartment, as well as partial or full thickness cartilage erosion of the femoral condyle, and to a lesser extent, in the tibial plateau. In most cases, there was little or no cartilage remaining in the MFC, leaving the subchondral bone exposed, which corresponded to the pathological changes in the terminal stage of human OA.

### Macroscopic grading in the middle and terminal stages of OA

**Fig. 2:**
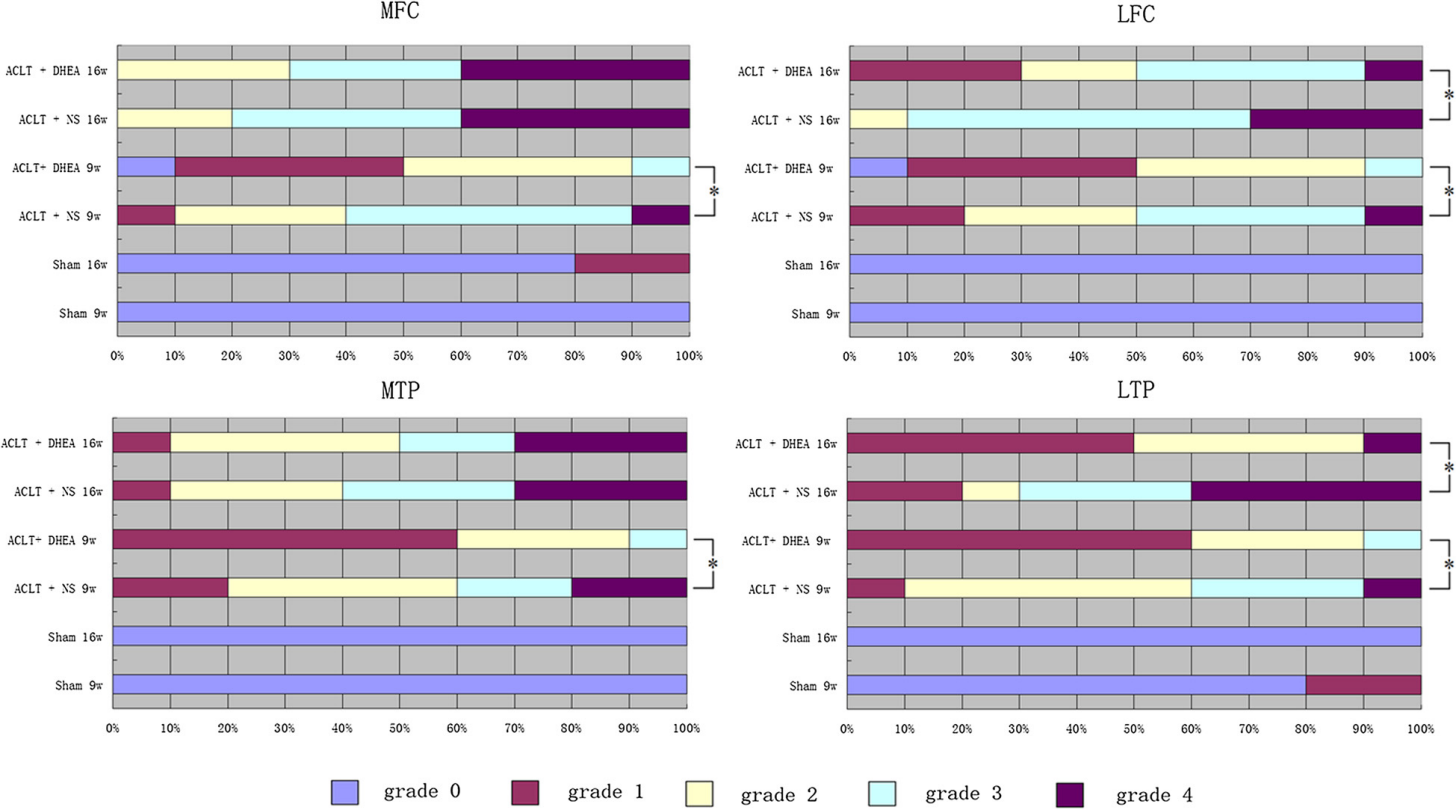
Macroscopic grades of the articular surfaces of the femoral condyles and tibial plateaus. Four compartments were graded (MFC, LFC, MTP and LTP). Data are presented as the mean percentage distribution of grades. *P < 0.05 for the DHEA-treated group compared with the placebo-treated group

**Fig. 3:**
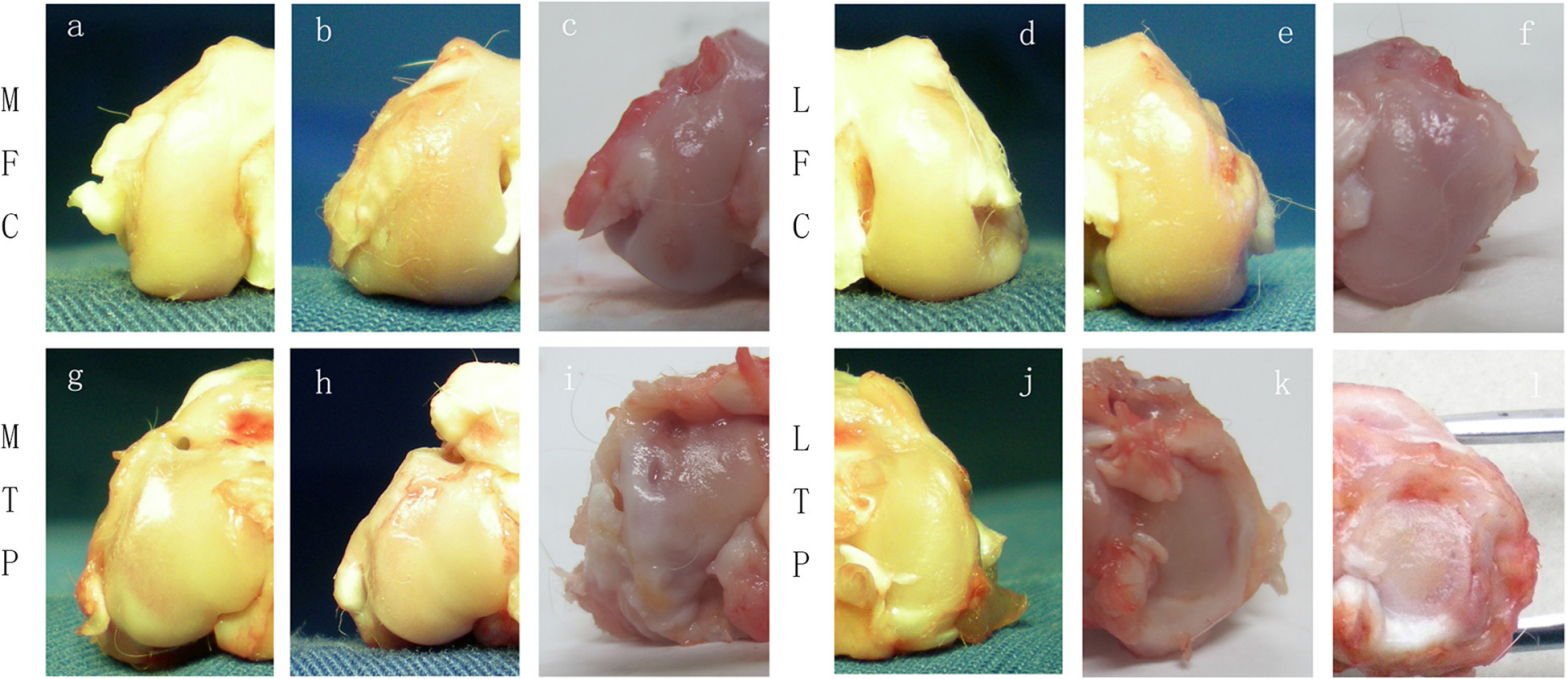
Typical macroscopic cartilage lesions among the groups in the four regions for the OA middle stage. The DHEA 9 w group exhibited a lower severity grade of macroscopic cartilage lesions in the 4 joint compartments compared with the NS 9 w group (P < 0.05). **a**-**c** represents the medial femoral condyles (MFC) of different groups (**a**: Sham, 9 w; **b**: ACLT+ DHEA, 9 w; **c**: ACLT + NS, 9 w). **d**-**f** represents the lateral femoral condyles (LFC) of different groups (**d**: Sham, 9 w; **e**: ACLT+ DHEA, 9 w; **f**: ACLT + NS, 9 w). **g**-**i** represents the medial tibial plateaus (MTP) of different groups (**g**: Sham, 9 w; **h**: ACLT+ DHEA, 9 w; **i**: ACLT + NS, 9 w). **j**-**l** represents the medial tibial plateaus (LTP) of different groups (**j**: Sham, 9 w; **k**: ACLT+ DHEA, 9 w; **l**: ACLT + NS, 9 w)

**Fig. 4:**
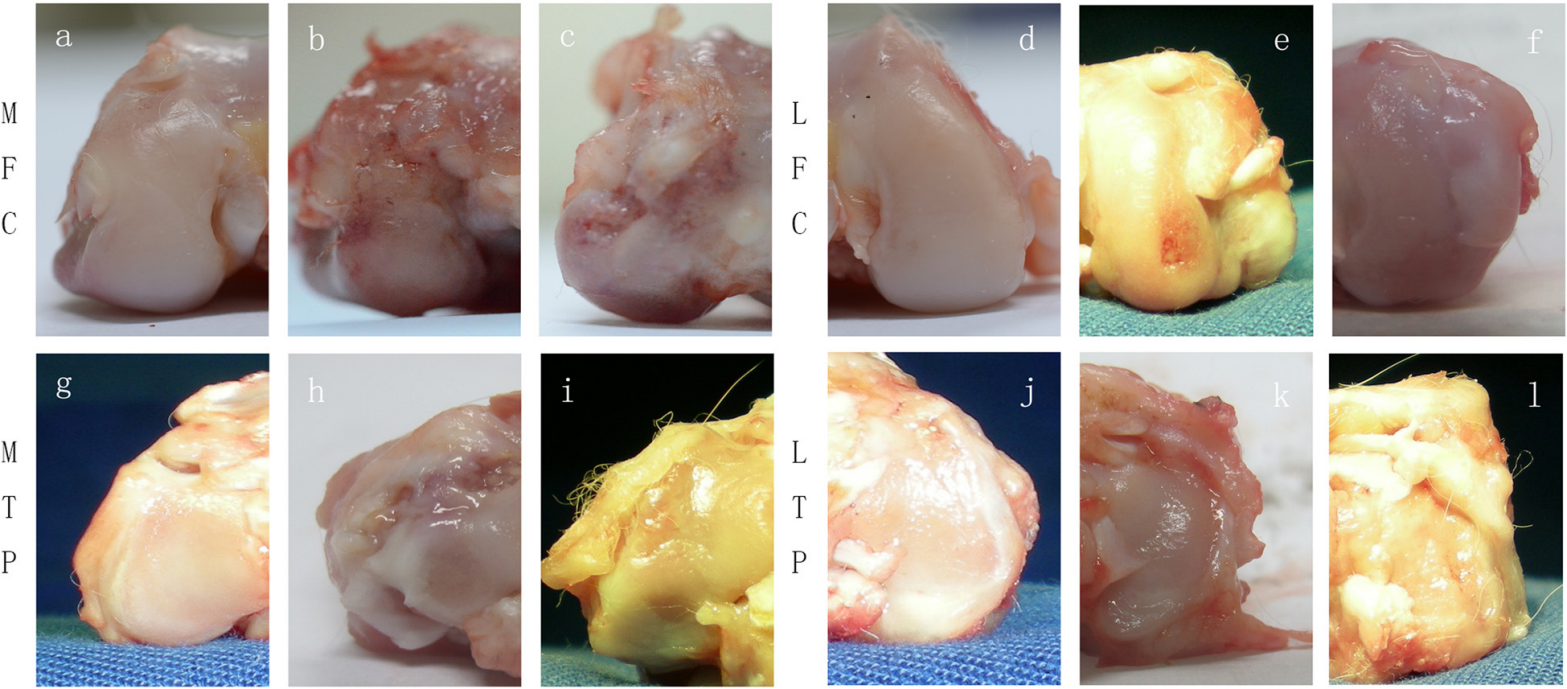
Typical macroscopic cartilage lesions among the groups in the four regions for the advanced stage of OA. The DHEA 16 w group exhibited significantly lower severity grades of macroscopic cartilage lesions in the LFC and LTP compartments compared with those observed in the NS 16 w group (P < 0.05); however, the between-group difference in the macroscopic scores for the MFC and MTP were not significant (P > 0.05). **a**-**c** represents the medial femoral condyles (MFC) of different groups (**a**: Sham, 16 w; **b**: ACLT+ DHEA, 16 w; **c**: ACLT + NS, 16 w). **d**-**f** represents the lateral femoral condyles (LFC) of different groups (**d**: Sham, 16 w; **e**: ACLT+ DHEA, 16 w; **f**: ACLT + NS, 16 w). **g**-**i** represents the medial tibial plateaus (MTP) of different groups (**g**: Sham, 16 w; **h**: ACLT+ DHEA, 16 w; **i**: ACLT + NS, 16 w). **j**-**l** represents the medial tibial plateaus (LTP) of different groups (**j**: Sham, 16 w; **k**: ACLT+ DHEA, 16 w; **l**: ACLT + NS, 16 w)

In the sham operation joints, the macroscopic grades were 0 (normal). No cartilage erosion was observed in any joint compartment. The sham group was included for comparative purposes to demonstrate that the ACLT surgery we performed induced degenerative changes in the articular cartilage. The DHEA 9 w group had a lower severity grade of macroscopic cartilage lesions in the 4 joint compartments compared with that in the NS 9 w group, and this difference was significant (MFC P = 0.013; LFC P = 0.048; MTP P = 0.045; LTP P = 0.02). The DHEA 16 w group has a significantly lower severity grade of macroscopic cartilage lesions in the LFC and LTP compartments compared with that in the NS 16 w group (LFC P = 0.046; LTP = 0.034); however, the between-group difference in the macroscopic score for the MFC and MTP was not significant (P > 0.05). Figure 2 presents the macroscopic grading in the four regions among the groups. Figures 3 and 4 display typical macroscopic cartilage lesions among the groups in the four regions for the different OA stages.

### Histological observations in the middle and terminal stages of OA

**Fig. 5:**
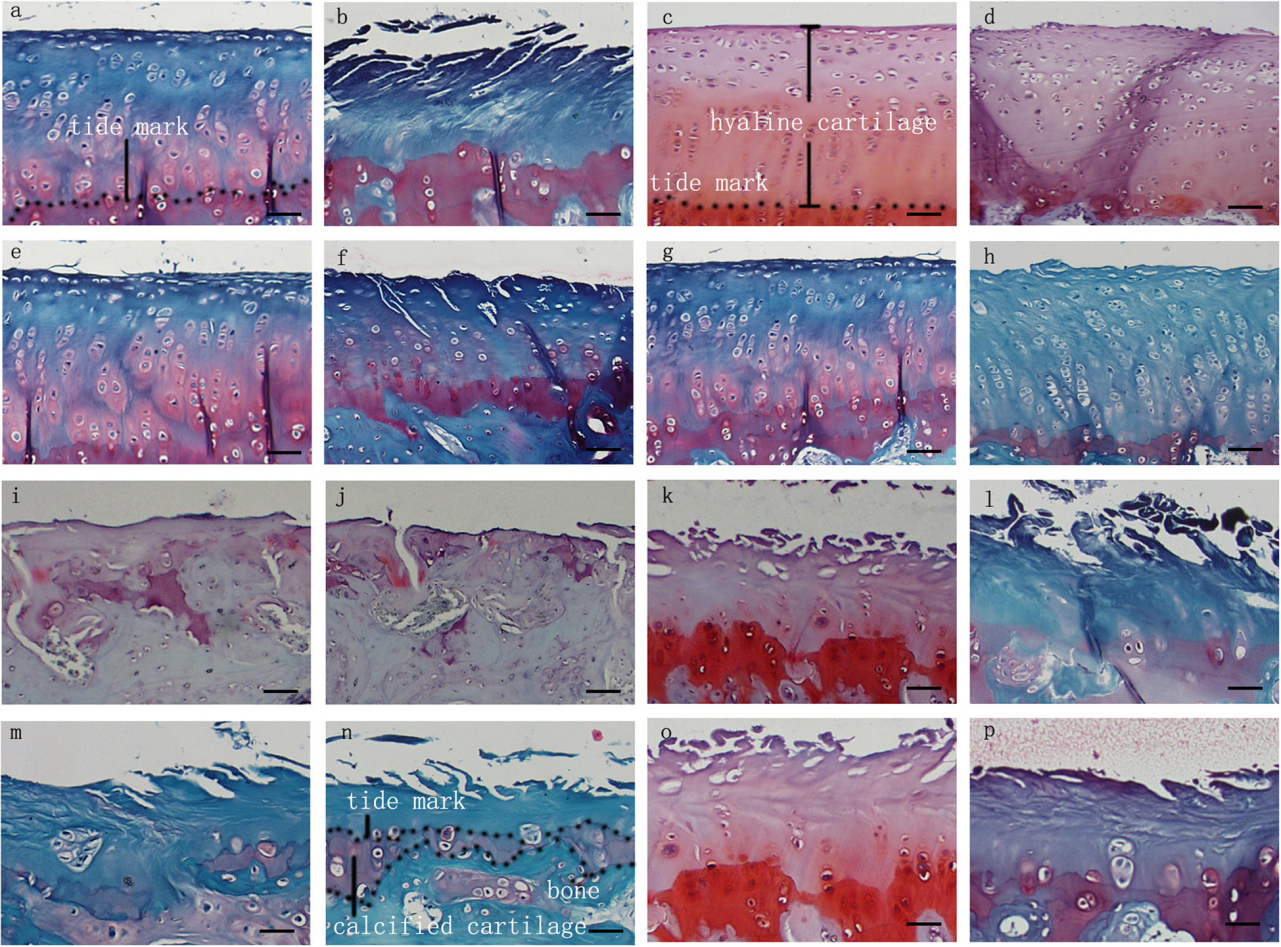
Representative Safranin O/fast green-stained histologic sections illustrating cartilage lesions. **a**-**h** represents histological changes among the groups in the four regions for the middle stage of OA. All DHEA-treated groups exhibited lower severity grades for histological cartilage lesions in the 4 joint compartments compared with placebo-treated groups with the exception that no histological differences for the MTP and MFC compartments were observed between the DHEA 16 w and NS 16 w groups. (**a**: MFC ACLT+ DHEA, 9w; **b**: MFC ACLT + NS, 9 w; **c**: LFC ACLT+ DHEA, 9w; **d**: LFC ACLT + NS, 9 w; **e**: MTP ACLT+ DHEA, 9w; **f**: MTP ACLT + NS, 9 w; **g**: LTP ACLT+ DHEA, 9w; **h**: LTP ACLT + NS, 9 w). **i**-**p** represents histological changes among the groups in the four regions for the advanced stage of OA (**i**: MFC ACLT+ DHEA, 16 w; **j**: MFC ACLT + NS, 16 w; **k**: LFC ACLT+ DHEA, 16 w; **l**: LFC ACLT + NS, 16 w; **m**: MTP ACLT+ DHEA, 16 w; **n**: MTP ACLT + NS, 16 w; **o**: LTP ACLT+ DHEA, 16 w; **p**: LTP ACLT + NS, 16 w). (Original magnification ×200, scale bar 50 μm)

**Fig. 6:**
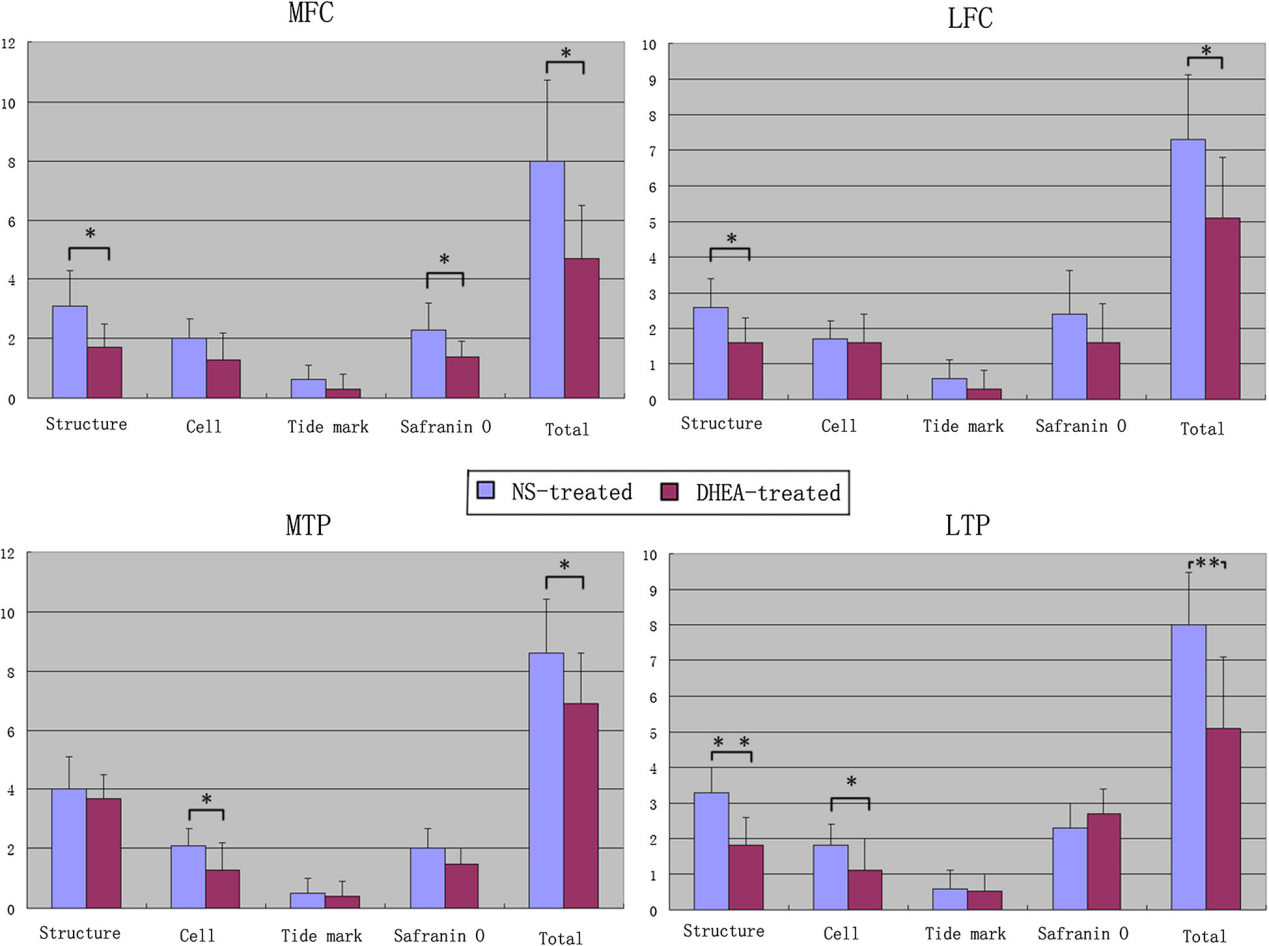
Histologic scores of the articular surfaces of the femoral condyles and tibial plateaus 9 weeks after operation. The scores for the histologic parameters with between-group comparisons are presented. Values represent the mean and SD. Histologic analysis revealed that the total Mankin score of DHEA-treated groups was significantly reduced compared with that of placebo-treated groups in both compartments of the knee. *P < 0.05 for the DHEA-treated group compared with the placebo-treated group. **P < 0.01 for the DHEA-treated group compared with the placebo-treated group

**Fig. 7:**
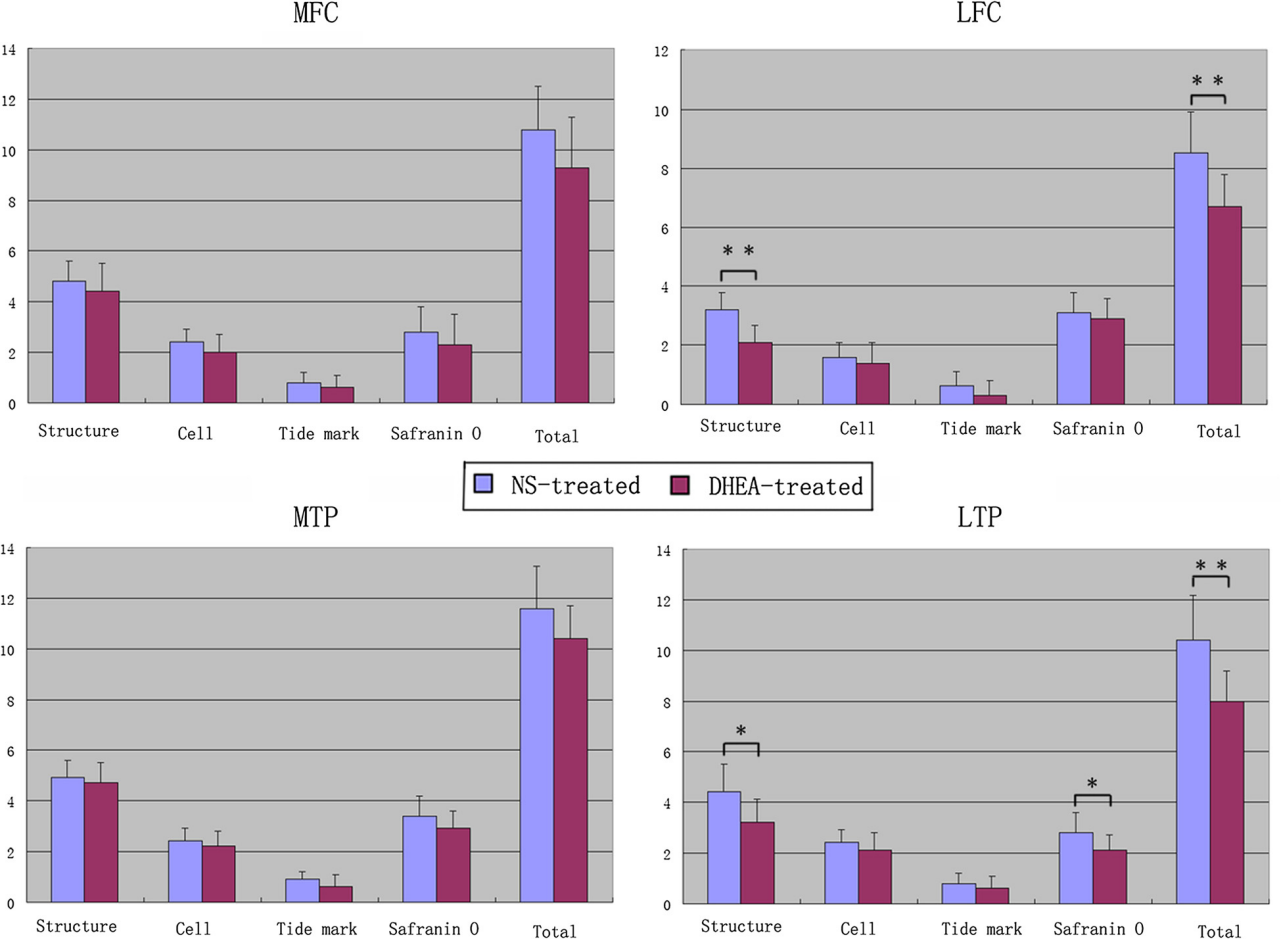
Histologic scores of the articular surfaces of the femoral condyles and tibial plateaus 16 weeks after operation. DHEA treatment reduced the total Mankin score in the lateral compartment. However, the total Mankin score of the medial compartment between groups does not have significant differenence. *P < 0.05 for the DHEA-treated group compared with the placebo-treated group. **P < 0.01 for the DHEA-treated group compared with the placebo-treated group

A quantitative histomorphometric assessment of the sections stained with Safranin O was performed using the Mankin scoring system. Similar to the degeneration trends in the macroscopic grading, the histological analyses showed that the lesions increased during middle to advanced OA. Typically, cartilage erosion with loss of the superficial or even the mid-zone was observed 9 weeks after ACLT. In the 16th week, microfractures became visible through the bone plate or the contour of the articular surface changed because of bone remodeling. Generally, minor lesions could be observed in the ACLT + DHEA group compared to the ACLT + NS group at 9 and 16 weeks after ACLT. In the DHEA groups, rare ulceration and a slight increase in the number of chondrocyte layers were observed. In addition, the osteophytes were smaller in the DHEA groups compared to those in the placebo groups. In the middle and advanced stages of OA, the cartilage was thicker in the DHEA groups than in the placebo groups. The distribution of chondrocytes in the areas with OA lesions was normal or slightly hypercellular in the DHEA groups, indicating regenerative activity; in the placebo groups, hypocellularity or aggregation of the chondrocytes was observed. Safranin-O staining, which could specifically bind to the proteoglycan in the ECM, was positive in the majority of the DHEA group specimens and in a minority of the placebo group specimens. The total Mankin scores of four regions were significantly higher in ACLT + NS group compared with those in ACLT + DHEA group at 9 weeks (MFC P = 0.012; LFC P = 0.034; MTP P = 0.016; LTP P = 0.002). The total Mankin scores of LFC and LTP in ACLT + NS group at 16 weeks is significantly higher than those in ACLT + DHEA group (LFC P = 0.005; LTP P = 0.002), while no statistically significant differences in the total Mankin scores for the MTP and MFC compartments were observed between the ACLT + DHEA 16 w and ACLT + NS 16 w groups which was consistent with the macroscopic data. The above data indicate that DHEA exerts an anabolic effect on cartilage by stimulating the synthesis of the cartilage matrix components and by increasing the proteoglycan synthesis mainly in the middle stage of OA. Figure 4 shows the quantitative histological results of the articular cartilage during the development of OA. Figure 5 shows typical sections of cartilage in different groups during both stages of OA. Figure 6 presents the histological score for articular cartilage of various groups 9 weeks after operation. Figure 7 presents the histological score for articular cartilage of various groups 16 weeks after operation.

## Discussion

OA is one of the leading causes of disability worldwide and is a serious burden to patients and society. Although the pathogenesis of OA is not fully understood, a central hallmark in this slow, chronic disease is progressive destruction of the articular cartilage. Thus, ideal medication should be able to retard this process and positively impact different aspects of the disease, including structural and metabolic changes in the joint components. DHEA appears to fulfill many of these requested criteria, such as having no toxic effects on the chondrocytes [9], possessing anti-inflammatory properties [14], and antagonizing the catabolic mediators and anabolic effects on the cartilage matrix [15].

The initial study by Williams et al. [16] reported that repeated administration of DHEA during arthritis induction delayed the onset and decreased the severity of collagen-induced arthritis in male and female DBA/1 mice. In a recent study, Jo et al. [17] showed that treating chondrocytes isolated from human osteoarthritic knee cartilage with DHEA significantly suppressed the gene expression and protein synthesis of MMP-1, while increasing the gene expression and protein synthesis of TIMP-1. Based on these studies, similar results in our previous work showed that DHEA protects against joint destruction and inhibits the progression of OA by modulating the catabolic/anabolic balance of cartilage, including the MMPs/TIMP-1 system [18] and the ADAMTS/TIMP-3 system [10, 19].

The aforementioned studies predominantly focus on the protective role of DHEA on cartilage at the molecular level. There is insufficient data demonstrating a potentially beneficial role of DHEA in the pathological changes that occur in OA. To provide a histological explanation for previous molecular results, this study focused on evaluating the histomorphological changes that occur in different joint compartments during the stages of OA after administering DHEA. The macroscopic and histological findings of this study matched well with the evidence provided in the above molecular experiments, indicating a chondroprotective role of DHEA in a surgically induced OA model.

The current data showed that the histological changes are time-dependent and site-specific; the cartilage lesion after ACLT showed increasing severity with the development of the disease and was similar to that in a clinical situation. In addition, the lesions in the medial femorotibial compartment presented earlier and were more advanced than those in the lateral compartment, likely because the medial compartment is the primary weight-loading region of the knee, thus the cartilage might degenerate faster than in other sections of the joint.

The Mankin analyses revealed that the DHEA injections effectively inhibited the progression of existing cartilage degeneration in both knee compartments at 9 weeks postoperatively and in the lateral knee compartment at 16 postoperative weeks. The Safranin O staining showed that the loss of proteoglycan could be prevented by DHEA administration, which is consistent with a relevant in vitro study reporting that DHEA could stimulate articular cartilage integration (reflected by increased glycosaminoglycan content) [20]. However, no effects were detected at 16 postoperative weeks in the MFC and MTP of the joint. This finding indicates that DHEA administration does not greatly affect the medial compartment, which endures the most stress force when OA develops into the advanced stage. We hypothesize that DHEA most likely plays a beneficial role when the cartilage matrix remains during disease progression, such as in the early and middle stages of OA. In the terminal stage of OA, full thickness cartilage damage occurs, leaving the subchondral bone exposed, particularly in the MFC and MTP, which causes the matrix restoration process conducted by the chondrocytes to be unresponsive to DHEA stimulation. Another explanation might be that in the terminal stage of OA, the cartilage degeneration process markedly exceeds the cartilage matrix recovery process induced by DHEA, leading to severe cartilage damage similar to that in the control group. Thus, the between-group difference in the cartilage quality in the medial compartment is not significant. We also found that the Mankin score of the lateral compartment of placebo group in terminal OA is significantly lower than that of medial compartment (data not shown). Based on the above two hypotheses, it is reasonable to explain why DHEA still work on the lateral compartment at advanced stage of OA.

Many studies have revealed the important role of the Wnt/β-catenin signaling pathway in the pathogenesis of OA [21–23]. Dell'accio and his colleagues [24] also demonstrated that Wnt/β-catenin proteins are up-regulated in joint areas with moderate-to-severe OA damage. Consistent with Dell'accio’s study, Lories et al. [25] also reported that cartilage loss is associated with a trend towards increased β-catenin levels in damaged cartilage in a mice model of OA. The results of the two studies suggest that Wnt/β-catenin signaling activation is enhanced in advanced OA with severe joint damage compared with the early and middle stages of the disease. Moreover, our most recent study [26] demonstrated that DHEA likely exerts its anti-osteoarthritic effect by regulating Wnt/β-catenin signaling. The protective effect of DHEA was significantly decreased when Wnt/β-catenin signaling was activated, whereas the inactivation of Wnt/β-catenin signaling enhanced the effects of DHEA. Thus, the third explanation demonstrates why DHEA administration does not greatly affect the medial compartment in the present research; in this region, the cartilage exhibited the most severe damage, and the activation of Wnt/β-catenin signaling is up-regulated.

Now osteoarthritis is viewed as a multifactorial disease affecting the whole joint. Although previous research focused primarily on changes in the articular cartilage, more recent studies have highlighted the importance of the subchondral bone, menisci, ligaments, synovium and periarticular muscles [27, 28]. Thus, a limitation of this study is that apart from articular cartilage we have not examined any effects of DHEA on other joint tissues like subchondral bone and menisci due to the limited joint specimen.

## Conclusion

Intra-articular DHEA injection plays a chondroprotective role in both compartments of the knee in the middle stage of OA, whereas DHEA was chondroprotective only in the lateral compartment in advanced OA. The disease-modifying efficacy of DHEA aganist OA is time-specific and site-dependent. DHEA could be used as a disease-modifying strategy to limit the progression of OA, especially in the middle stage.

## Abbreviations

ACLT: Anterior cruciate ligament transaction
DHEA: Dehydroepiandrosterone
OA: Osteoarthritis
MFC: Medial femoral condyle
LFC: Lateral femoral condyle
MTP: Medial tibial plateau
LTP: Lateral tibial plateau
NS: Normal saline
